# The Impact of Maternal Spinal Anesthesia on Newborn Out-Comes: A Clinical Perspective

**DOI:** 10.3390/children12040450

**Published:** 2025-03-31

**Authors:** Ramona Celia Moisa, Nicoleta Negrut, Iulia Codruta Macovei, Cristina Aur, Mihai Octavian Botea, Paula Bianca Maghiar, Cezar Cristian Mihai Moisa, Harrie Toms John, Paula Marian

**Affiliations:** 1 Clinic of Anaesthesia and Intensive Care, Pelican Clinic, Medicover Hospital, 4104869 Oradea, Romania; moisa.ramona.celia@didactic.uoradea.ro (R.C.M.); mbotea@uoradea.ro (M.O.B.); 2 Doctoral School of Biomedical Sciences, Faculty of Medicine and Pharmacy, University of Oradea, 410087 Oradea, Romania; badea.paula.bianca@didactic.uoradea.ro; 3 Department of Surgery, Faculty of Medicine and Pharmacy, University of Oradea, 410087 Oradea, Romania; cmacovei@uoradea.ro (I.C.M.); d.aur@uoradea.ro (C.A.); 4 Department of Psycho-Neuroscience and Recovery, Faculty of Medicine and Pharmacy, University of Oradea, 410073 Oradea, Romania; 5 Faculty of Medicine and Pharmacy, University of Oradea, 410087 Oradea, Romania; moisa.cezarcristianmihai@student.uoradea.ro; 6 Department of Intensive Care, Epsom and St. Helier University Hospitals NHS Trust, Wrythe Lane, Sutton, Carshalton SM5 1AA, UK; harrie.tomsjohn@uoradea.ro; 7 Department of Medical Disciplines, Faculty of Medicine and Pharmacy, University of Oradea, 410073 Oradea, Romania; paulamarian@uoradea.ro

**Keywords:** spinal anesthesia, morphine, fentanyl, Apgar

## Abstract

Background/Objectives: Spinal anesthesia, frequently used in cesarean deliveries, can have a significant impact on newborns. This study aims to evaluate the effects of spinal anesthesia with morphine or fentanyl as adjuvants on neonatal outcomes. Methods: A retrospective study was conducted over a specific period on 170 newborns delivered via cesarean section at the Pelican Clinic, Medicover Hospital, Romania. The neonatal parameters assessed included Apgar scores at 1 and 5 min, oxygen saturation, respiratory rate, and heart rate in two groups of newborns whose mothers underwent spinal anesthesia with bupivacaine combined with either morphine or fentanyl (group M_n and group F_n). Statistical analysis was performed using IBM SPSS Statistics (version 29.0.2.0 (20)). Results: Newborns in the M_n group had significantly higher Apgar scores at 1 min compared to those in the F_n group (9.63 ± 0.57 vs 9.40 ± 0.65, *p* = 0.010); however, at 5 min, the scores were comparable between groups. Regarding oxygen saturation, male neonates born to mothers who received morphine had significantly higher values than those in the fentanyl group (96.08 ± 4.14% vs. 94.50 ± 4.36%, *p* = 0.026), whereas no significant differences were observed in female neonates. Conclusions: The use of morphine in maternal spinal anesthesia may improve immediate neonatal adaptation, particularly in male newborns.

## 1. Introduction

Spinal anesthesia is widely used in obstetric surgery, providing a safer and more effective alternative to general anesthesia. The current medical literature highlights multiple benefits of spinal anesthesia, including cardiorespiratory stability, rapid postoperative recovery, and a lower mortality risk [1,2]. In addition to maternal safety, spinal anesthesia has been associated with a lower incidence of neonatal resuscitation, higher Apgar scores, lower admission rates to neonatal intensive care units, and prompt restoration of gastrointestinal function in newborns [3,4].

Despite its advantages, this technique is not risk-free. Maternal hypotension during the procedure may lead to transient fetal metabolic acidosis due to reduced uteroplacental perfusion [5]. The choice of intrathecal adjuvants can further influence neonatal adaptation. These agents have been associated with neonatal respiratory depression, fetal sedation, and delayed initiation of breastfeeding, especially at higher doses [6,7]. Additional neonatal manifestations may include transient tachypnea, difficulty with pulmonary adaptation, particularly in elective cesarean sections performed before labor onset, and changes in neonatal heart rate, with bradycardia or tachycardia [8,9].

While spinal anesthesia is a safe and effective option for cesarean deliveries, with the potential to improve neonatal outcomes and reduce maternal complications, the decision regarding the type and dosage of anesthetic agents should be individualized [4].

Morphine and fentanyl are intrathecal adjuvants commonly used in cesarean deliveries, each with distinct pharmacological profiles that may differentially influence neonatal clinical parameters. Morphine, a hydrophilic opioid, provides strong and prolonged spinal analgesia, with effects lasting up to 24 h after cesarean delivery but with a relatively slow onset of approximately 30 min following administration [10,11]. Fentanyl is highly lipophilic, resulting in a rapid onset of analgesia within minutes but a much shorter duration of action, typically between 1 and 4 h [12]. These pharmacokinetic differences also impact placental transfer rates: fentanyl crosses the placenta more easily, with reported fetal-to-maternal plasma concentration ratio of 0.89, which may lead to earlier neonatal effects [13]. In contrast, morphine shows a lower transfer rate, typically below 0.3, which may reduce such risks [14].

Although intrathecal opioids, such as morphine and fentanyl, are widely used in spinal anesthesia for cesarean delivery, direct comparative studies evaluating their specific effects on neonatal outcomes remain limited. Most available evidence focuses on maternal analgesia or reports general neonatal risks without differentiating between agents. Moreover, potential differences in neonatal response based on sex, particularly regarding oxygen saturation, an important marker of early adaptation, have been largely underexplored. Addressing these gaps is essential for understanding whether pharmacological differences between opioids translate into clinically relevant neonatal effects.

Therefore, this study aims to evaluate the impact of maternal spinal anesthesia on immediate neonatal outcomes based on the type of intrathecal opioid used during cesarean delivery. Additionally, we investigate whether neonatal sex influences these effects, with a particular focus on oxygen saturation. Our goal is to provide evidence-based guidance for optimizing spinal anesthesia practices in obstetric care. We hypothesize that the type of opioid adjuvant used in spinal anesthesia (morphine or fentanyl) differentially influences immediate neonatal adaptation, as reflected in the Apgar scores, oxygen saturation levels, and cardiorespiratory parameters.

## 2. Materials and Methods

### 2.1. Study Design

The study retrospectively examines data on newborns delivered via cesarean section from pregnant women admitted to Clinica Pelican, Medicover Hospital, Romania, between 1 January 2023, and 30 June 2024. This study is part of a broader doctoral thesis and has received approval from the University of Oradea’s Ethics Committee (no. 2084/13 February 2025). Additionally, it adheres to the ethical principles of the World Medical Association’s Declaration of Helsinki (2024). Informed consent was obtained from all parturient women at the time of their admission to the hospital.

**Figure 1:**
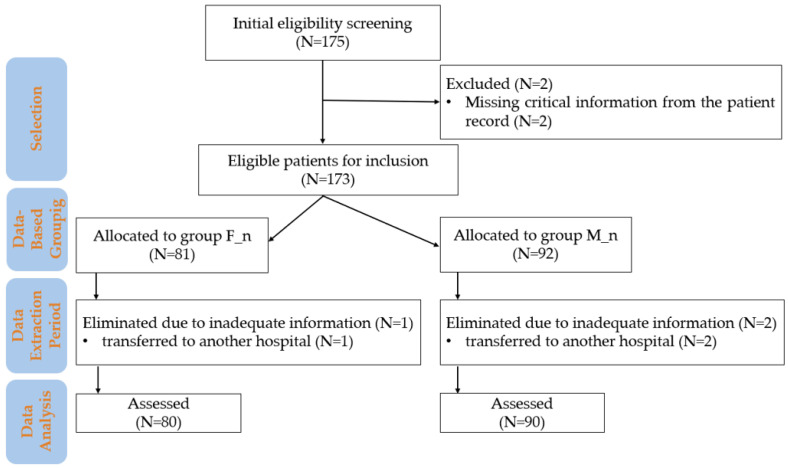
Flowchart of patient inclusion and data analysis in the study. M_n—newborns from mothers with morphine anesthesia; F_n—newborns from mothers with fentanyl anesthesia; n—number.

The study included subjects based on pre-established eligibility criteria without active allocation interventions. The inclusion and exclusion criteria used in the study are presented in Figure 1.

### 2.2. Inclusion Criteria

- Maternal age between 18–50 years.
- Singleton pregnancies without significant complications.
- Planned cesarean delivery under spinal anesthesia (administration of M or F).
- Term pregnancy between 37–42 weeks of gestation.
- No major congenital anomalies.
- Complete clinical data for both mother and newborn.
- Informed consent signed by the mother.

### 2.3. Exclusion Criteria

- Maternal history of chronic conditions.
- Severe pregnancy complications (e.g., preeclampsia or placenta previa).
- Prematurity (<37 weeks of gestation).
- Severe neonatal conditions at birth.
- Use of general or combined anesthesia.
- Use of unconventional techniques or substances in anesthesia.
- Lack of relevant information in the files.
- Transferred to another hospital.

### 2.4. Maternal Anesthetic Protocol

Spinal anesthesia was performed according to the current Romanian clinical guidelines and the standardized protocol of our hospital, which includes the routine use of intrathecal opioids for cesarean sections to optimize postoperative pain management [15,16].

As stipulated by hospital protocol, all parturient women received pre-anesthetic treatment consisting of 500 mL of lactated Ringer’s solution, 40 mg of pantoprazole, and 10 mg of metoclopramide.

Spinal anesthesia was performed via intrathecal administration of hyperbaric bupivacaine 5 mg/mL (7.5–11 mg, adjusted according to the subject’s height), combined with a solution of morphine 1 mg/mL (fixed dose of 100 µg) or fentanyl 50 µg/mL (fixed dose of 25 µg). Maternal hypotension was managed with repeated administration of ephedrine solution 5 mg/mL (5–15 mg per dose), as needed.

### 2.5. Data Collection

Newborn parameters were monitored after birth to evaluate any potential influences of maternal anesthesia on neonatal status.

The attending neonatologist evaluated the Apgar score (AS) at 1 (_1_) and 5 (_5_) minutes based on the following clinical criteria: heart rate, respiratory effort, muscle tone, reflex irritability, and skin color. Each criterion was scored from 0 to 2, resulting in a total score ranging from 0 to 10. The Apgar scores were assessed by experienced neonatologists, all of whom are part of the hospital’s neonatal team, following standardized clinical protocols. Although multiple neonatologists were involved, all were trained in uniform procedures. The environmental conditions (temperature and lighting) in the delivery room were maintained at constant levels, and all cesarean sections were performed during the hospital’s daytime operating hours, from 8 a.m. to 4 p.m.

The neonatal ventricular rate (VR_n) and neonatal respiratory rate (RR_n) were measured immediately after birth.

The neonatal peripheral capillary oxygen saturation (SpO_2_n) was measured immediately after birth by placing a pulse oximetry sensor on the newborn’s right hand. Preductal (SpO_2_pre) and postductal (SpO_2_post) oxygen saturations were recorded 24 h after birth, by placing pulse oximetry sensors on the newborn’s right hand and foot, respectively.

The following devices were used for neonatal monitoring. A Masimo Rad-97 pulse oximeter (Masimo Corporation, Irvine, CA, USA) was used to measure the VR_n, SpO_2_n, SpO_2_pre, and SpO_2_post. This device provides real-time heart rate [beats per minute (bpm)] and oxygen saturation.

A GE Healthcare-adapted monitor (General Electric, Chicago, IL, USA) was used to measure the RR_n, providing real-time respiratory rate [breaths per minute (brpm)]. A digital stopwatch (iPhone, Apple Inc., Los Altos, CA, USA) measured the time from spinal anesthesia to baby extraction (TSA-BE). The measurement started immediately after the intrathecal injection of the anesthetic solution and stopped at the complete extraction of the neonate from the uterine cavity.

This study is part of a broader research project investigating maternal and neonatal parameters. A separate study analyzing the maternal aspects has been submitted for publication [15].

### 2.6. Statistical Analysis

The data were analyzed statistically using IBM SPSS Statistics (version 29.0.2.0 (20)). Descriptive statistics were used to evaluate the demographic and clinical characteristics of the subjects in the two study groups.

The normality of the distribution of continuous variables was assessed using the Shapiro–Wilk test, with subsequent adaptation of comparison tests for each dataset. For parameters with a non-normal distribution, the Mann–Whitney U test was applied, while the Kruskal–Wallis test was used to compare mean ranks among groups defined by the administered ephedrine dose. The correlations between the ephedrine dose and neonatal parameters were evaluated using the Spearman correlation coefficient (ρ). Fisher’s exact test was applied to compare the distribution of bradycardia (BC) and tachycardia (TC) cases between groups, while the Pearson Chi-square test was used for the distribution of categorical data. ANOVA analysis evaluated the impact of anesthesia type and sex on SpO_2_n values.

All statistical tests were performed with a significance threshold of *p* < 0.05.

## 3. Results

### 3.1. Clinical and Demographic Characteristics

The clinical and demographic characteristics of the subjects included in the study are presented in Table 1.

### 3.2. Apgar Score

**Figure 2:**
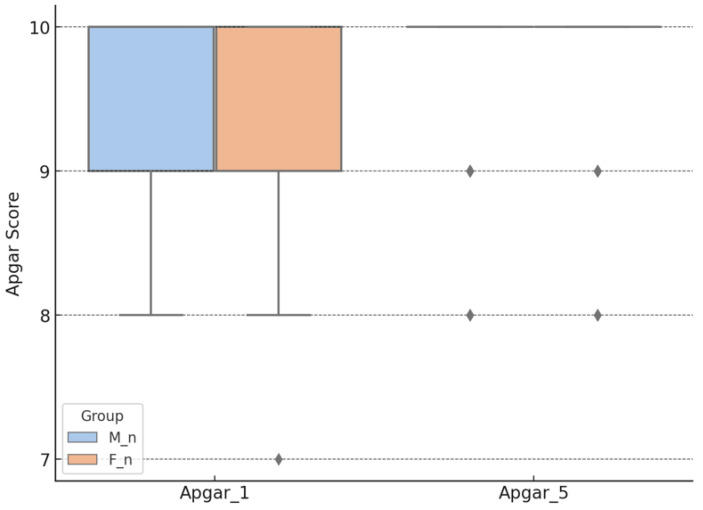
Apgar score, according to the type of anesthesia. M_n—newborns from mothers with morphine anesthesia; F_n—newborns from mothers with fentanyl anesthesia; Apgar_1—Apgar score at 1 min after birth; Apgar_5—Apgar score at 5 min after birth.

The Apgar_1 score was 9.63 ± 0.57 for the M_n group and 9.40 ± 0.65 for the F_n group. The Apgar_5 score was 9.88 ± 0.39 in the M_n group and 9.90 ± 0.38 in the F_n group. All groups exhibited a non-normal distribution of data (Shapiro–Wilk test, *p* < 0.001). The Mann–Whitney test for the group comparisons indicated a significant difference at 1 min (*p* = 0.010) but not at 5 min (*p* = 0.581), as shown in Figure 2.

Ordinal logistic regression analysis did not show a significant impact of ephedrine on the Apgar score, regardless of whether it was administered in combination with morphine or fentanyl, both at 1 min (*p* = 0.779 for the direct effect of ephedrine and *p* = 0.867 for its interaction with the anesthesia) and at 5 min (*p* = 0.816 and *p* = 0.773, respectively). Furthermore, no significant differences were observed between fentanyl and morphine in their influence on the Apgar score at 1 min (*p* = 0.081) and 5 min (*p* = 0.857), indicating that neither ephedrine nor the type of opioid used has a relevant effect on this neonatal parameter, as shown in Table 2.

### 3.3. Neonatal Ventricular Rate and Respiratory Rate

**Figure 3:**
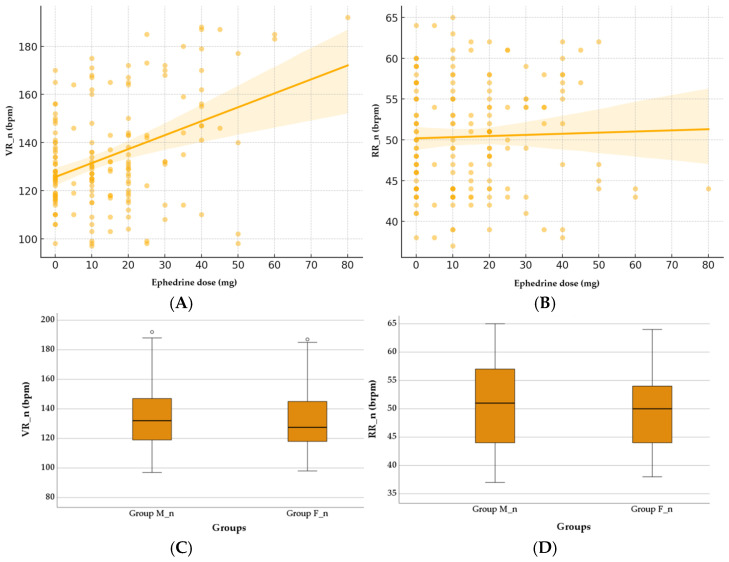
Effect of ephedrine and anesthesia type on neonatal VR_n and RR_n. (**A**) Correlation between ephedrine dose and neonatal VR_n. (**B**) Correlation between ephedrine dose and neonatal RR_n. (**C**) Comparison of neonatal VR_n between Group M_n and Group F_n. (**D**) Comparison of neonatal RR_n between Group M_n and Group F_n. Group M_n—group of newborns from mothers with morphine anesthesia; Group F_n—group of newborns from mothers with fentanyl anesthesia; VR_n (bpm)—neonatal ventricular rate (beats per minute); RR_n (brpm)—neonatal respiratory rate (breaths per minute).

Neonatal physiological responses were not significantly influenced by the ephedrine dose or the anesthesia type. The relationship between the ephedrine, VR_n, and RR_n was explored, as shown in Figure 3. Spearman’s correlation test did not reveal a significant association between the ephedrine dose and the VR_n (ρ = 0.03, *p* = 0.708) or the RR_n (ρ = −0.04, *p* = 0.685), and the scatter plots (Figure 3A,B) confirm this lack of a clear trend. The boxplots (Figure 3C,D) indicate a tendency for the VR_n to decrease in the F_n group compared to the M_n group (133.25 ± 22.52 vs. 135.71 ± 22.43, *p* = 0.292, Mann–Whitney U Test), which is consistent with the nearly significant trend observed in the regression analysis (coefficient = −8.29, *p* = 0.085). In contrast, the RR_n does not show significant differences between anesthesia types (F vs. M) (50.07 ± 6.50 vs. 50.67 ± 6.98, *p* = 0.534, Mann–Whitney U Test).

According to Fisher’s exact test, as shown in Table 3, the analysis of the distribution of BC and TC cases in the two groups with different anesthesia types did not reveal statistically significant differences (*p* = 1.0, *p* = 1.0).

### 3.4. Peripheral Capillary Oxygen Saturation

**Figure 4:**
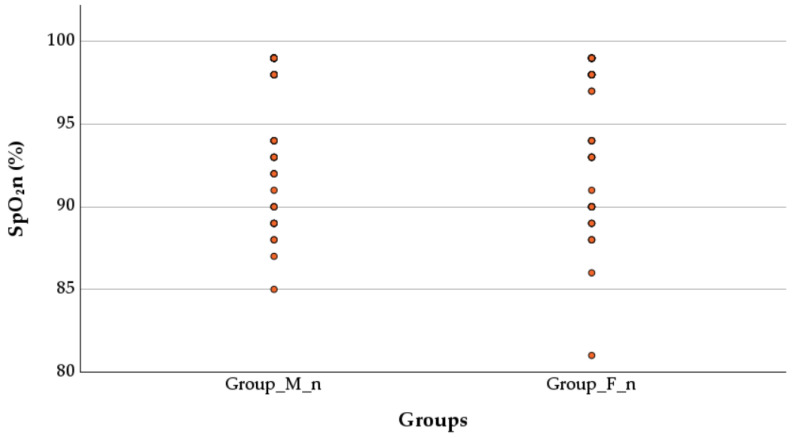
Effect of anesthesia type on peripheral capillary oxygen saturation. SpO_2_n—neonatal peripheral capillary oxygen saturation; Group M_n—group of newborns from mothers with morphine anesthesia; Group F_n—group of newborns from mothers with fentanyl anesthesia.

The SpO_2_n values after birth in newborns were non-significantly higher in the M_n group compared to the F_n group (96.11 ± 3.94 vs. 95.45 ± 4.28, *p* = 0.115, Mann–Whitney test), as shown in Figure 4.

**Figure 5:**
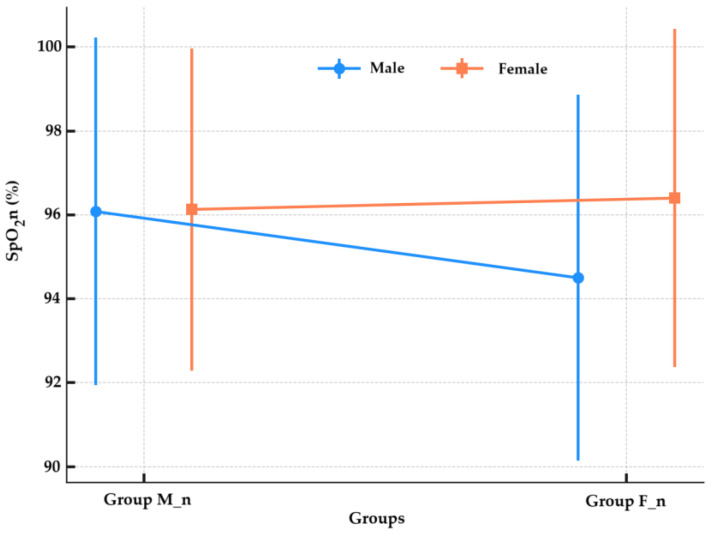
Oxygen saturation by anesthesia group and sex. SpO_2_n—neonatal peripheral capillary oxygen saturation; Group M_n—group of newborns from mothers with morphine anesthesia; Group F_n—group of newborns from mothers with fentanyl anesthesia.

The SpO_2_n values were 96.08 ± 4.14% in male neonates from the group M_n (n = 37) and 96.13 ± 3.84% in girls from the same group (n = 53). For the group F_n, the SpO_2_n was 94.50 ± 4.36% in male neonates (n = 40) and 96.40 ± 4.03% in girls (n = 40). Male neonates in group M_n had significantly higher SpO_2_n values than those in group F_n (Mann–Whitney U test, *p* = 0.026). In girls, there was no significant difference between the two anesthesia types (Mann–Whitney U test, *p* = 0.803), as shown in Figure 5. The analysis of the proportion of newborns with SpO_2_ values below 96% in both groups was performed, and no statistically significant difference between group F_n (31, 38.75%) and group M_n (31, 34.44%) (Chi-Square test, *p* = 0.673) was found. The proportion of male newborns with SpO_2_ < 96% was higher in the group F_n (19/31, 61.29%) compared to the group M_n (12/31, 38.71%), but this difference did not reach statistical significance (Chi-Square test, *p* = 0.128).

## 4. Discussion

Spinal anesthesia proves useful in cesarean deliveries because it ensures cardio-vascular stability and better maternal and neonatal outcomes. The decision between anesthetic drugs and vasopressor treatment affects how well neonates adjust following birth, yet additional research is required to validate these practices.

### 4.1. Apgar Score

Neonatal adaptation occurs immediately following birth, combined with maternal anesthesia, fetal oxygen quality, and natural system regulation, to provide an overall perception of the Apgar score. Intrathecal morphine was associated with a statistically higher 1-min Apgar score; however, the mean difference was slight (0.23 points), and both values fall well within the normal clinical range, thus limiting its clinical significance. Nonetheless, the convergence of scores at 5 min indicates a rapid compensatory adjustment, regardless of the anesthetic used. The study conducted by Karaman et al. (2011) on two groups similar to the present study, but with samples of 20 participants per group, supports that the Apgar scores at 1 and 5 min after birth do not differ based on the adjuvant used for the mother’s spinal anesthesia [17]. Alkaya Solmaz et al.’s (2016) study of 60 patients undergoing elective cesarean section found no statistically significant differences in Apgar scores at 1 and 5 min based on the intrathecal adjuvant (morphine or fentanyl) used for spinal anesthesia [18]. At the 5-min mark, both anesthetic agents demonstrated comparable neonatal recovery, while the initially observed differences in the Apgar_1 score may be explained by the rapid placental transmission of fentanyl [19]. However, the discrepancies observed in this study may be partially explained by the larger sample size compared to the previously cited study.

The analysis of ordinal data demonstrated that ephedrine medication given to mothers did not impact their neonates’ first (*p* = 0.779) and fifth (*p* = 0.540) Apgar scores, irrespective of whether they received fentanyl or morphine during anesthesia. The systematic review by Lee et al. found that administering ephedrine during spinal anesthesia for cesarean delivery did not significantly affect the Apgar scores at 1 and 5 min when compared to the controls [20].

### 4.2. Neonatal Ventricular Rate and Respiratory Rate

The VR_n and RR_n are influenced by maternal autonomic function, catecholamine release, and placental perfusion, all of which can be affected by the choice of spinal anesthesia and vasopressor administration. In the present study, ephedrine was administered in response to maternal hypotension as part of the standard management protocol. Therefore, any potential impact on neonatal outcomes should be interpreted in the context of maternal hypotension rather than as a direct pharmacological effect of ephedrine. The lack of significant differences in the VR_n and RR_n between the groups and the absence of a correlation with the ephedrine dose indicates a limited impact of the anesthetic agent on these specific physiological responses during spinal anesthesia. To our knowledge, this is the first study to evaluate this aspect. Excessive doses can induce tachycardia due to maternal sympathetic stimulation. The lack of significant differences in the current study may be due to the controlled administration of ephedrine and the avoidance of extreme doses that could alter fetal heart rate variability.

### 4.3. Peripheral Capillary Oxygen Saturation

The SpO_2_n depends on developmental lung status, oxygen diffusion ability, and blood circulatory stability, yet these factors show potential sensitivity to delivery anesthesia choices. The trend toward higher neonatal oxygen saturation in the morphine group did not reach statistical significance overall; however, subgroup analysis revealed a significant advantage in male neonates, pointing toward a potential interaction between neonatal sex and the type of intrathecal opioid administered. To the best of our knowledge, this is one of the first studies to specifically investigate the combined effect of maternal spinal anesthesia type and neonatal sex on SpO_2_n values immediately post-birth. The observed differences in SpO_2_n measurements between male and female neonates may result from natural physiological differences between the sexes, including variations in lung development and hormonal impacts. This finding suggests that female neonates exhibit similar oxygenation responses to varying anesthesia types, resulting in equivalent SpO_2_n measurements. Additional research with extended and heterogeneous participant sample sizes should perform complete physiological assessments to establish conclusive associations in this field.

Neonatal oxygen saturation in the immediate postpartum period is a sensitive indicator of perinatal adaptation and may be influenced by the type of maternal anesthesia. In the present study, although no statistically significant differences were observed between groups, a higher proportion of male newborns exposed to intrathecal fentanyl showed oxygen saturation values below the 96% threshold. This finding could reflect subtle sex-specific differences in respiratory or neurohumoral adaptation to birth, potentially modulated by opioid pharmacodynamics. To our knowledge, this is the first study to explore such associations in the context of intrathecal anesthesia for cesarean delivery. One possible explanation could be sex-based differences in neonatal pulmonary adaptation or opioid receptor sensitivity. While data on sex-specific responses to intrathecal anesthesia are limited, sex differences in lung development and neuroendocrine regulation could underlie this observation. Further research is needed to confirm and explore these findings.

This study is part of a doctoral thesis, which includes a separate analysis focusing on maternal parameters [15].

### 4.4. Limitations and Practical Implications

The current study has several limitations. The retrospective design and single-center setting may limit the generalizability of the results. Although statistically significant sex-related differences in neonatal oxygen saturation were observed, the study was not designed to support robust subgroup analysis. Furthermore, the analysis was limited to immediate neonatal outcomes, with no follow-up of long-term respiratory or neurodevelopmental outcomes. Confounding variables, such as maternal age, parity, gestational age at birth, and TSA-BE, were not included in the multivariate regression models. Future prospective studies with larger, multicenter cohorts are needed to confirm these findings.

## 5. Conclusions

The present study indicates that administering fentanyl or morphine during maternal spinal anesthesia may influence neonatal clinical parameters immediately after birth. Newborns of mothers who received morphine as an adjuvant in spinal anesthesia had significantly higher Apgar scores at 1 min compared to those whose mothers received fentanyl; however, these differences were no longer observed at 5 min postnatally. Additionally, oxygen saturation immediately after birth was significantly higher in male newborns of mothers who received morphine compared to those whose mothers received fentanyl, while no notable differences were observed in female neonates. Although overall rates of lower oxygen saturation did not differ significantly between groups, a higher proportion of male neonates in the fentanyl group showed suboptimal SpO_2_ values, suggesting a possible sex-related sensitivity to the type of intrathecal opioid used.

These findings suggest a potential advantage of morphine over fentanyl in terms of specific immediate neonatal parameters; however, the clinical relevance is limited, and further prospective research is needed to validate these preliminary observations.

## Figures and Tables

**Table 1:** Characteristics of the study groups.

Parameter	Group F_n (n = 80)	Group M_n (n = 90)	*p*-Value
DD			
Urban residence, n (%)	55 (68.8)	62 (68.9)	0.984 ^##^
Female, n (%)	40 (50.0)	53 (58.89)	0.314 ^##^
Clinical data			
TSA-BE, sec., M ± SD	449.03 ± 74.45	439.46 ± 67.80	0.545 ^#^
SpO_2_pre (right hand) ≥ 95%, n (%)	80 (100)	90 (100)	1 ^##^
SpO_2_pre (right hand), %, M ± SD	98.04 ± 0.81 (96, 99)	97.98 ± 0.77 (95, 99)	0.499 ^#^
SpO_2_post (foot) ≥ 95%, n (%)	80 (100)	90 (100)	1 ^##^
SpO_2_post (foot), %, M ± SD	98.59 ± 0.56 (97, 99)	98.66 ± 0.58 (96, 99)	0.318 ^#^
ΔSpO_2_ ≤ 3%, n (%)	80 (100)	90 (100)	1 ^##^
ΔSpO_2_, %, M ± SD	−0.55 ± 0.84 (−3, 1)	−0.68 ± 0.74 (−2, 1)	0.246 ^#^

DD—demographic data; TSA-BE—time from spinal anesthesia to baby extraction; sec.—seconds; SpO_2_pre (right hand)—preductal oxygen saturation; SpO_2_post (foot)—postductal oxygen saturation; ΔSpO_2_—preductal-postductal difference; n—number; M—mean; SD—standard deviation; ^#^—Mann-Whitney U Test; ^##^—Pearson Chi-Square.

**Table 2:** Influence of the maternally administered ephedrine dose on the Apgar_1 and Apgar_5 scores.

Parameter Estimates								
		Estimate	SE.	Wald	df	*p*-Value	95% CI	
							Lower Bound	Upper Bound
Threshold	Fentanyl_Morphine	−0.778	0.447	3.035	1	0.081	−1.653	0.097
	[Apgar_1 = 7]	−5.655	1.049	29.063	1	<0.001	−7.710	−3.599
	[Apgar_1 = 8]	−3.405	0.461	54.476	1	<0.001	−4.309	−2.501
	[Apgar_1 = 9]	−0.794	0.328	5.852	1	0.016	−1.438	−0.151
Location	Dose of ephedrine	−0.004	0.013	0.079	1	0.779	−0.030	0.022
	Epinephrine_anesthesia type_interaction	−0.004	0.021	0.028	1	0.867	−0.045	0.038
Threshold	Fentanyl_Morphine	0.140	0.779	0.032	1	0.857	−1.386	1.666
	[Apgar_5 = 8]	−3.679	0.674	29.818	1	<0.001	−5.000	−2.359
	[Apgar_5 = 9]	−2.287	0.521	19.277	1	<0.001	−3.308	−1.266
Location	Dose of ephedrine	−0.005	0.020	0.054	1	0.816	−0.045	0.035
	Epinephrine_anesthesia type_interaction	0.011	0.039	0.084	1	0.773	−0.065	0.088

SE—Standard Error; CI—confidence interval; Wald—Wald Chi-Square Statistic; df—degrees of freedom; Apgar_1—Apgar score at 1 min after birth; Apgar_5—Apgar score at 5 min after birth.

**Table 3:** Characteristics of the study groups.

Parameter	Group F_n	Group M_n	*p*-Value
BC, n (%)	3 (3.75%)	4 (4.44%)	1 ^#^
TC, n (%)	14 (17.50%)	15 (16.67%)	1 ^#^

Group M_n—group of newborns from mothers with morphine anesthesia; Group F_n—group of newborns from mothers with fentanyl anesthesia; BC—bradycardia; TC—tachycardia; ^#^—Fisher’s exact test.

## Data Availability

The datasets presented in this article are not readily available because they are part of an ongoing study. Requests to access the datasets should be directed to Nicoleta Negrut.
